# Correlation between changes in intraocular pressure and refractive error indices post Cataract Surgery

**DOI:** 10.12669/pjms.36.3.1597

**Published:** 2020

**Authors:** Muhammad Afzal Bodla, Ali Afzal Bodla, Ayema Moazzam, Noor Tariq

**Affiliations:** 1 Muhammad Afzal Bodla, DORCS. Department of Ophthalmology, Multan Medical and Dental College Multan, Pakistan; 2 Ali Afzal Bodla, FRCS. Department of Ophthalmology, Multan Medical and Dental College Multan, Pakistan; 3 Ayema Moazzam, Department of Ophthalmology, Multan Medical and Dental College Multan, Pakistan; 4 Noor Tariq, Department of Ophthalmology, Multan Medical and Dental College Multan, Pakistan

**Keywords:** Cataract surgery, Intraocular pressure, Phacoemulsification, Refractive errors

## Abstract

**Objective:**

To evaluate the correlation between refractive errors and change in intraocular pressure in patients undergoing cataract surgery.

**Methods:**

This interventional retrospective case study was carried out from September 2018 to April 2019 at Bodla Eye Care and Multan Medical and Dental College, Multan. A total of 127 eyes were recruited in the study among which six were excluded. Out of remaining 121, 53 eyes were emmetropes, 41 were mild myopes and 27 were high myopes. Single surgeon performed the procedure. Pre-operative investigations of IOP and refractive error were done by goldmann tonometry and auto refractometry. IOP was reviewed at day 1, 7, 14 and 28 post cataract surgeries.

**Results:**

Out of 121 eyes, 53 eyes were emmetropes, 41 were mild myopes and 27 were high myopes, who underwent phacoemulsification. There was an elevation of 2-3mm Hg at Day-1, in emmetropes and mild myopes, and further on, a constant drop was noticed on follow ups. In high myopes a significant fluctuation of IOP was noted in first fourteen days followed by an unremarkable gradual decline afterwards.

**Conclusion:**

Cataract surgery helps lowering the IOP in patients with refractive errors. Mild myopic and emmetropic patients showed a linear swift pattern while high myopes presented instable and gradual reduction in IOP. A total decrease of 1-2mm Hg was seen at the end of the study depicting that relation between IOP and cataract surgery is insignificant.

## INTRODUCTION

Cataract is an opacification in the lens of the eye interfering with visual functions,^1^,^2^ with it being the cause of 39% of blindness and 33% of severe visual impairments across the world whereby the pervasiveness in age-related cataract cases seems to be increasing. For years the cataract surgery has been performed and is still manifested beneficial in visual improvement.^3^ Cataract surgery was suggested to decrease intraocular pressure (IOP) in the eyes with or without glaucoma, in addition to removing the opacified lens.^4^,^5^ and after the success of cataract surgery it has therefore been preferred for glaucoma patients.^6^

Various elements that affect the intraocular pressure in patients after a cataract surgery have been studied.^7^ Sufficient information indicates that cataract surgery has a long-term reduction impact on IOP. This effect appears to be proportional to preoperative IOP. Eyes with greater preoperative IOP have the largest average decrease, whereas eyes with IOP in the reduced range of statistically normal range tend to have an IOP that stays unchanged from baseline or maybe higher after cataract surgery.

In patients with narrow angles, the IOP-lowering effect appears to be proportional to the degree of anterior chamber deepening induced by cataract surgery.^8^ Various refractive errors have subsequent effects on Intraocular Pressure, post cataract; myopia for instance, being the most common cause.

However, Myopia, the most common type of refractive error, is a complex trait including both genetic and environmental factors^9^ Progressive myopia can lead from the sclera’s hereditary biomechanical weakness that enables the sclera to stretch in reaction to stress. The objective of this study was to determine whether there is any significant lowering of intraocular pressure post cataract surgery in normotensive patients. Moreover, authors are inclined to determine whether a specific refractive error contributes more to this phenomenon.

## METHODS

This was an interventional retrospective case analysis of 127 eyes that went through cataract surgery in Bodla Eye Care and Multan Medical and Dental College carried out from September 2018 to April 2019 (Ref. MMDC-, dated September 4, 2018). Six of them being excluded because of set inclusion criteria were patients of age 20 years & older, willing and able to understand and sign consent form and attend post-operative follow-ups as per schedule. These patients should have been free of any other ophthalmic disease, e.g. glaucoma, ARMD etc. Candidates with hypermetropia, ocular hypertension, systemic diseases, eye diseases other than cataract such as glaucoma, conditions affecting IOP and those who underwent extracapsular cataract extraction (ECCE) were excluded. Informed written consent was obtained from all subjects who participated in this study. The confidentiality of the participants was maintained for the study. The data was collected by four follow-up sessions including preoperative visit to the surgeon. Sample included patients who were myopic, were diagnosed of cataract and received phacoemulsification. Surgeries were performed by same surgeon (Bodla, AA) and all the materials were kept same to avoid disparity. Patient’s bio-data (age, sex and general physical examination), preoperative examination (refractive error via Auto Refraction and Intraocular Pressure via goldmann tonometry) and postoperative examinations (refractive conditions and intraocular pressure on day one, seven, 14 and 28 were noted. Refractive error were classified 0 to -0.25D as emmetropia, -0.5D to -5.75D as mild to moderate myopia and -6 & greater as high myopia. Analysis was done using SPSS software version 20.0. Data was evaluated using descriptive statistics and relation with variables was calculated via Chi-square test. A value P<0.2 in the analysis was considered as a variable selection criterion.

## RESULTS

One hundred twenty-one eyes underwent phacoemulsification at Bodla Eye Care & MMDC in period of September, 2018 – April, 2019, out of which ‘53’ were emmetropes, ‘41’ were mild-moderate myopes & ‘27’ were high myopes. They all embraced the criteria that were set. Surgeon performing surgery (Bodla, AA) & intraocular lens implanted were kept same. IOP was statistically insignificant in relation to the refractive errors. (P=0.011).

IOP at Day 1 in patients with emmetropia and mild myopia, after surgery was slightly elevated but (P=0.105) not significant (pre-operative being 16.3±3.3). IOP on further follow ups at Day 7 (13.6±4.4), Day 14 (13.2±3.2) & Day 28 (12.9±2.8) was significantly lower with (P<0.001). This depicted that IOP was decreasing after surgery.

In high myopia, we found a higher IOP at day one and lower IOP at 7 and 14 days compared with IOP before surgery with no statistic difference (*P*=0.49, *P=* 0.57, and *P=* 0.13,) With a considerably reduced 28-day IOP than before surgery (P < .001), respectively. And no significant difference was found between day 1 and 7, 7 and 14 days, and 14 and 28 days (*P=*0.423, *P=*0.739, and *P=*0.359, respectively).

Myopic and emmetropic patients showed almost same trend as mentioned above. In high myopes, IOP rose at day one & decreased in follow-up sessions but didn’t reach the statistical significant values. Among the various groups of refractive errors, high myopes had raised IOP in comparison to others. The changes in IOP at day 1,7,14 and 28 are shown in Table-I.

**Table-I:** Correlation between refractive errors and change in IOP.

Refractive error	IOP at day 1	IOP at day 7	IOP at day 14	IOP at day 28
Emmetropes	16.3±3.3	13.6±4.4	13.2±3.2	12.9±2.8
Mild Myopes	15.8±2.7	14.4±2.3	13.9±1.9	13.2±1.5
High Myopes	15.2±2.9	16.4±2.6	14.6±2.8	13.1±1.8

## DISCUSSION

In this study, we have traversed the relationship between various refractive conditions to post-surgical IOP. We found that cataract surgery with phacoemulsification and intra ocular lens implantation resulted in 87% success for achieving the goal of decreased IOP.^10^

In a previous study^11^ similar to this, it was depicted that IOP is reduced in post cataract surgeries in glaucoma as well as non-glaucoma patients, due to deepening in the anterior chamber by implantation of IOL, however, which not was congruous to our study.An elevation of 3-4mm Hg was noticed in the immediate post-operative period, it was also well demarcated in previous studies that there is an elevation initiated at three hours and peaked after 24 hours.^12^,^13^ In a relevant study by Bonnell et al., a significant elevation (10-20mm Hg) in IOP 24 hours post-operative was narrated.^14^ In comparison, our study exhibited a slight increase in the IOP (3-4mm Hg) at day one that might be due to post-operative inflammation reaction and blockage of trabecular meshwork^15^ by viscoelastic and left out cortical material.^16^,^17^ Furthermore, emmetropes and mild myopes showed almost same results. There was a linear decrease of IOP in first 14 days as analyzed by change in IOP in these groups and remained persistent for upcoming days. A total of 2-3mm Hg was however reduced which was found to be insignificant.

In candidates with high myopia, there wasn’t any manifest change in IOP up till third follow up, after which an instable decline was seen. The reason behind indifferent values of IOP in high myopes might be due to anatomical change that includes raise in visual axis^18^, thinning of choroid and retina, enlarged optic disc and peri-papillary atrophy.^19^ These manifestations exist even after phacoemulsification, thus giving contradictory effects to post-operative lowering of IOP. This phenomenon is the evidence of greater tendency of post-operative IOP fluctuation in high myopia patients that was in harmony to previous studies^20^ and this may be the risk factor for worsening of glaucoma.^21^

### Limitations of the study

This study shows the relationship between phacoemulsification and post-operative change in IOP. Our study has few limitations. Firstly, protocol of evaluation of chamber angle configuration was missing. Secondly, due to non-cooperation of few patients, follow up data was lost. Thirdly, being retrospective analysis, we lacked proper data. In synopsis, cataract surgery has an impact on IOP irrespective of the refractive conditions.

## CONCLUSION

Our analysis inferred that the emmetropic, mild to moderate myopic and high myopic patients that underwent phacoemulsification showed depression in IOP at the end of trial follow up, depicting that cataract surgery decreases the IOP regardless of refractive conditions with postoperative time elongation. Hence, cataract surgery does leads to decrease in IOP irrespective of refractive error, though changes are insignificant.

### Author`s Contribution:

**MAB** formulated, designed and did editing of manuscript.

**AAB, AM and NT** did data collection, statistical analysis and manuscript writing.

**AAB** did review, final approval of manuscript and is responsible for integrity of research.
